# Neural correlates of the self-reference effect: evidence from evaluation and recognition processes

**DOI:** 10.3389/fnhum.2015.00383

**Published:** 2015-06-26

**Authors:** Ken Yaoi, Mariko Osaka, Naoyuki Osaka

**Affiliations:** ^1^Department of Psychology, Graduate School of Letters, Kyoto UniversityKyoto, Japan; ^2^Department of Psychology, Graduate School of Human Sciences, Osaka UniversityOsaka, Japan

**Keywords:** self-reference effect, medial prefrontal cortex, episodic memory, encoding, retrieval

## Abstract

The self-reference effect (SRE) is defined as better recall or recognition performance when the memorized materials refer to the self. Recently, a number of neuroimaging studies using self-referential and other-referential tasks have reported that self- and other-referential judgments basically show greater activation in common brain regions, specifically in the medial prefrontal cortex (MPFC) when compared with nonmentalizing judgments, but that a ventral-to-dorsal gradient in MPFC emerges from a direct comparison between self- and other-judgments. However, most of these previous studies could not provide an adequate explanation for the neural basis of SRE because they did not directly compare brain activation for recognition/recall of the words referenced to the self with another person. Here, we used an event-related functional magnetic resonance imaging (fMRI) that measured brain activity during processing of references to the self and another, and for recognition of self and other referenced words. Results from the fMRI evaluation task indicated greater activation in ventromedial prefrontal cortex (VMPFC) in the self-referential condition. While in the recognition task, VMPFC, posterior cingulate cortex (PCC) and bilateral angular gyrus (AG) showed greater activation when participants correctly recognized self-referenced words versus other-referenced words. These data provide evidence that the self-referenced words evoked greater activation in the self-related region (VMPFC) and memory-related regions (PCC and AG) relative to another person in the retrieval phase, and that the words remained as a stronger memory trace that supports recognition.

## Introduction

People normally prioritize the perception and processing of self-related stimuli over stimuli unrelated to the self. Like the “cocktail party effect,” our name or other self-related information strongly attracts our attention and becomes relatively better processed. Prior cognitive functions bias our memory for self-referenced items, a phenomenon known as the self-reference effect (SRE; Greenwald and Banaji, 1989). SRE is defined as better recall or recognition performance when the memorized materials refer to the self. For example, when participants judge words as descriptive of the self, they recall or recognize them better than had they described others. To date, various hypotheses about the cognitive mechanisms of SRE have been proposed (Symons and Johnson, 1997). For example, Rogers et al. (1977) suggested that self-reference judgments produce a “rich” encoding unit that can function effectively during information processing. Furthermore, Bellezza (1984) argued that SRE occurs because the self provides a set of organized internal cues, and the materials associated with the cues are easier to retrieve during recognition/recall tasks. They indicated that recall of the internal cues generated during learning is necessary for the recall of the presented material and that a single-factor theory for encoding processes is insufficient to explain the recall results found in the self-reference task compared with those found in various control conditions. Symons and Johnson (1997) reviewed many SRE studies and indicated that this effect is primarily because the self is a well-developed and often-used construct in memory, promoting both the elaboration and organization of encoded information. These studies suggested that self-reference is a relatively special process in encoding the evaluation phase, in on the retrieval of the recognition/recall phase or both.

A number of neuroimaging studies have tried to clarify the neural correlates of self-referential processing and whether it depends upon a “unique” neural basis. Recently, however, many researchers claimed that self- and other-referential judgments basically show greater activation in common brain regions, specifically in the medial prefrontal cortex (MPFC) when compared with nonmentalizing judgments, for example, counting words length (Schmitz et al., 2004; Legrand and Ruby, 2009; Mitchell, 2009; Spreng et al., 2009; Yaoi et al., 2009; Denny et al., 2012). On the other hand, Denny et al. (2012) performed a meta-analysis of neuroimaging studies and reported that there is a ventral-to-dorsal gradient in MPFC for self- to other judgments from a direct comparison between both. Furthermore, other reviews or meta-analyses reached approximately the same conclusion (van der Meer et al., 2010; Qin and Northoff, 2011; Wagner et al., 2012).

However, most imaging studies that used the self-referential task only investigated the encoding process for evaluation tasks and not the neural correlates of the retrieval process (in recognition or recall tasks) for self-referenced materials. Philippi et al. (2012) found that in patients with lesions to MPFC, SRE is eliminated but that SRE is normal in patients with lesions to other brain regions and in healthy adults. They suggested that MPFC is necessary for SRE and is important for self-referential processing and a neural representation of the self. This result is consistent with neuroimaging studies that have pointed to the importance of MPFC in the self-referential (and perhaps also in the other referential) process (Kelley et al., 2002; Macrae et al., 2004; Schmitz et al., 2004; Moran et al., 2006; Northoff et al., 2006). However, because MPFC lesions in patients would affect throughout the task (from encoding to the retrieval phase), it is difficult to determine in which phase (encoding, retrieval, or both) MPFC plays an important role for SRE.

Few previous studies that investigated the neural correlates of SRE during encoding or retrieval (Fossati et al., 2004; Macrae et al., 2004; Benoit et al., 2010; Gutchess et al., 2010; Leshikar and Duarte, 2012, 2014) recognized the involvement of the ventral part of MPFC. For example, Macrae et al. (2004) examined normal subjects using the self-reference task in an event-related functional magnetic resonance imaging (fMRI) and set the contrast according to whether the referenced word was later remembered or forgotten and whether the item was judged as self-relevant. They found that activity in MPFC predicted both subsequent memory performance and judgment of self-relevance. However, they did not discuss the difference between correct-recognition and forgotten processes in the recognition task for the self-referenced words. Furthermore, Leshikar and Duarte (2012) measured neural activity while participants studied and subsequently retrieved pictures of common objects under either self-reference or self-external encoding instructions. They suggested that processing information in relation to the self leads to mnemonic benefits for source level features, and that activity in MPFC contributes to this source memory benefit. Recently, Leshikar and Duarte (2014) investigated the effects of self-reference on source memory performance and associated neural activity in young and older adults. They suggested that the encoding phase activity in the dorsal part of MPFC supports source memory accuracy for self-referenced materials similarly in both age groups. However, none of these works focused on the difference between self- and other-referential processing from the standpoint of MPFC activation in the recognition phase. Thus, we still have many questions regarding how self-referenced items are retrieved/recalled better than other and whether or not MPFC activation is different in the recognition phase. To clarify the cognitive and neural basis of SRE, it appears necessary to investigate the neural correlates of both encoding and recognition/recall processes, and to compare the self with other targets in the experiment.

In a recent study, Morel et al. (2014) measured brain activity under the self, other person, and control semantic conditions during the encoding and recognition phases. They specifically focused on the differences between the self and the semantic control condition and indicated that the self-referential condition showed greater activity in MPFC and hippocampus. They also found greater functional coupling between those brain regions and the posterior cingulate cortex (PCC) during the encoding phase only. SRE is shown as a comparison of self-reflection accessing a basic level of semantic knowledge; however, there are also comparisons of the self with other people, especially those distant from the participant (Symons and Johnson, 1997; Yaoi et al., 2009). Therefore, we assumed that there were some cognitive and neural differences between the self and other conditions during the encoding or retrieval phase.

In the present study, we focused on the differences between the self and other conditions and hypothesized that self-referenced items are involved in special cognitive properties that require specific processes and corresponding neural activities for the retrieval of items, relative to other referenced items. For example, if the self is constituted of a rich and organized representation that acts as an internal cue in the retrieval process as Bellezza (1984) pointed out, self-representation may (re)activate when retrieving in a recognition (or recall) task rather than others. Therefore, to investigate the neural correlates of SRE, we measured brain activity during the processing of references to the self and others and of recognition to the self and other referenced words using event-related fMRI with specific interest in regions like MPFC.

## Materials and Methods

### Subjects

Twenty-three right or corrected-to-right-handed and one left-handed healthy Japanese participants were recruited (males *n* = 14, mean age = 23.3 ± 4.0 years). All participants had normal or corrected-to-normal vision and reported no history of psychiatric or neurological disorders. The experiments were conducted in accordance with the ethical guidelines of the Brain Activity Imaging Center (ATR, Kyoto, Japan). All participants provided written, informed consent prior to the experiments. Four participants did not respond to 5% or more of all trials and one participant showed 100% correct answers in the self-recognition task. These five participants were excluded from the analysis, and all behavioral and neuroimaging analyses were conducted on the remaining 18 participants only.

### Materials

Three hundred Japanese trait adjectives were selected from the corpus of Aoki (1971), who examined the desirability ratings of 455 words. All trait adjectives were written with two to nine characters in Japanese. From these 300 words, we selected the top 100 high-desirability (2.0–4.3) words and bottom 100 low-desirability (6.2–7.9) words. Each 100 word list was divided into four groups (25 high- or 25 low-desirability words) as their average desirability and word lengths were nearly equal. We then paired a high- and a low-desirability group and made four word lists (including 25 high- and 25 low-desirability words). Two of the four word lists (50 high- and 50 low-desirability words) were used in the evaluation task, and the other two word lists were used as fillers for the recognition task. We counterbalanced the allocation of the word lists across participants. For a preliminary practice task, another six words were selected from 155 words.

### fMRI Tasks

**Figure 1:**
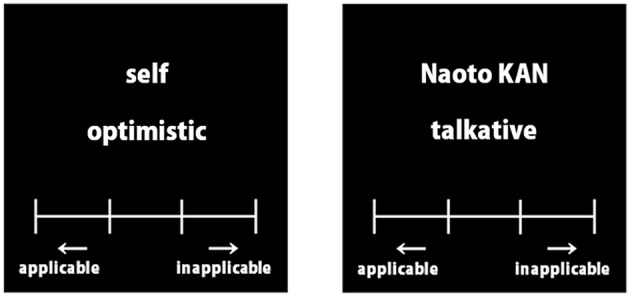
**Example words of the evaluation task**. Each trait adjective was presented in Japanese.

To investigate brain activities during the processing of referential information relating to either the self or a distant other, we employed a rapid event-related design paradigm similar to that of Kelley et al. (2002). In the self-referential condition, participants were required to judge how well they thought each trait adjective described them. Similarly, in the other referential condition, participants were required to imagine Naoto Kan (a previous Japanese prime minister), and judge how well each word described him. In these two conditions, participants chose one of four alternatives, “almost never,” “rarely,” “sometimes,” or “almost always” using four buttons to declare their decision (Figure 1). One or two of the above-mentioned word lists was assigned for the self- and other-referential conditions.

After the evaluation task, participants rested outside the fMRI scanner for about 30 min. They then returned inside to the scanner and performed the recognition task. Here participants were required to respond “old” or “new” to 200 words using buttons; 100 words had been used in the evaluation task (“old”) and 100 were distractors (“new”). Because we aimed to investigate the incidental learning of words used in the evaluation task, participants were not informed about this task prior to the experiment.

#### Apparatus

While in the MR scanner, the participant held in each hand an fMRI response device connected to a computer via an optic cable. Each of the two hand devices had two buttons (Current Designs, Inc., Philadelphia, PA, USA) and participants had to press one of the four buttons on the keypad using the index or middle finger in the evaluation task. In the recognition task, participants had one response device in their right hand and were required to press one of the two buttons using the thumb.

#### Procedure

Prior to the main task (and after the preliminary task), participants performed a practice task. Using a procedure similar to that of the main task, each condition contained six words consisting of three self-referential and three other-referential conditions.

In the evaluation task, each word remained on the screen for 4000 ms. Participants were instructed to evaluate each word by pressing a response button and they were allowed to judge at any time within the 4000 ms. Afterward, small fixation crosshairs were presented for variable (jittered) and inter-trial-interval (ITI) durations that ranged from 4000 ms to 12,000 ms at the center of the screen. These stimuli were projected onto a mat screen and presented to the participants through a mirror. In the recognition task, each word remained on the screen for 2000 ms. The methods for defining other parameters were the same as for the evaluation task.

### Data Acquisition

Whole brain imaging data were acquired using a 3.0 Tesla MRI scanner (Siemens MAGNETOM Verio) and a standard head coil. Head movements were minimized with cushions at each side of the head. For functional imaging, we used a gradient-echo echo-planar imaging sequence with the following parameters: repetition *time*_(TR)_ = 2000 ms; echo *time*_(TE)_ = 30 ms; flip angle = 80°; field of *view*_(FOV)_ = 192 × 192 mm; and voxel size = 3 × 3 × 5 mm. After image collection, T1-weighted anatomical images using a conventional spin echo pulse sequence (TR = 2250 ms, TE = 3.06 ms, flip angle = 9°, FOV = 256 × 256 mm, and voxel size = 1 × 1 × 1 mm) were collected for anatomical co-registration at the same locations as the functional images. Scanner sequences were synchronized with the stimulus presentation using the stimulus software Presentation (Neurobehavioral Systems, Inc., Berkeley, CA, USA).

### Data Analysis

All data were analyzed using SPM 5 (Wellcome Department of Imaging Neuroscience, London, UK) on Matlab 7.3 (MathWorks, Inc., Natick, MA, USA). The first six images were discarded from the analysis to eliminate any non-equilibrium effects of magnetization. All functional images were realigned to correct for head movements, which were less than 2.0 mm within runs. Functional images were normalized and spatially smoothed with an isotropic Gaussian filter (8 mm full-width at half-maximum). Low-frequency noise was removed with high-pass filtering (128 s). For the evaluation task, we set four functional events and onsets (self_correct, other_correct, self_miss and other_miss), which were defined by whether the word presented in the evaluation task was correct-to-recognized in the recognition task. For the recognition task, we added another two events (correct rejection and false alarm), which were defined by whether each filler word in the recognition task was correct-to-rejected. These events were modeled with a gamma hemodynamic response function (HRF) that was applied when trait words in each condition appeared onscreen. Group data were analyzed using a random effects model. Activation areas for all conditions were specified at *p* < 0.05 cluster level family-wise error (FWE) corrected for multiple comparisons with the amplitude of voxels surviving at *p* < 0.001 uncorrected across the whole brain.

## Results

### Behavioral Data (Evaluation Task)

Table 1 shows behavioral performance (mean reaction time, RT) under each condition in the evaluation task. The four conditions were defined by whether the words referred to the self or other and recognized as correct or miss in the recognition task. For mean RTs during the evaluation task, a 2 (condition: self vs. other) × 2 (performance: correct vs. miss) ANOVA did not reveal a significant effect for either factor, that is, condition (*F*_(1,17)_ = 1.11, *p* > 0.05) and performance (*F*_(1,17)_ = 0.60, *p* > 0.05), but there was a significant interaction (*F*_(1,17)_ = 4.74, *p* < 0.05). Tukey’s honest significant difference *post hoc* analysis indicated that mean RTs during the self_correct were marginally faster than those during self_miss and other_correct (*p* < 0.10), but there were no significant differences between any conditions (*p* > 0.05).

**Table 1:** **Mean reaction time (RT) and standard deviation (*SD*) in the evaluation task**.

	Self_correct	Self_miss	Other_correct	Other_miss
*M* (ms)	2296.4	2404.7	2413.7	2390.3
*SD*	361.4	424.9	363.9	493.3

*Four word conditions (“self_correct, “self_miss, “other_correct, and “other_miss”) were used. Each word was categorized based on the correct-to-recognized result in the recognition task*.

### Behavioral Data (Recognition Task)

Table 2 shows mean recognition performance for the self- and other-referenced words in the recognition task. For mean recognition performance (self: 84.2%, *SD* = 10.5; other: 64.1%, *SD* = 16.9), a *t* test revealed significant differences between the self- and other-referenced words (*t*_(17)_ = 6.2, *p* < 0.001). This result indicates that words referring to the self were better recognized than those referring to other (SRE).

**Table 2:** **Percent correct recognition (M) and standard deviation (*SD*) for the self-referenced and other-referenced words, and mean percent and SD of false alarm (FA) in the recognition task**.

	Self	Other	FA
*M* (%)	84.2	64.1	19.3
*SD*	10.5	16.9	11.0

### fMRI Data (Evaluation Task)

**Figure 2:**
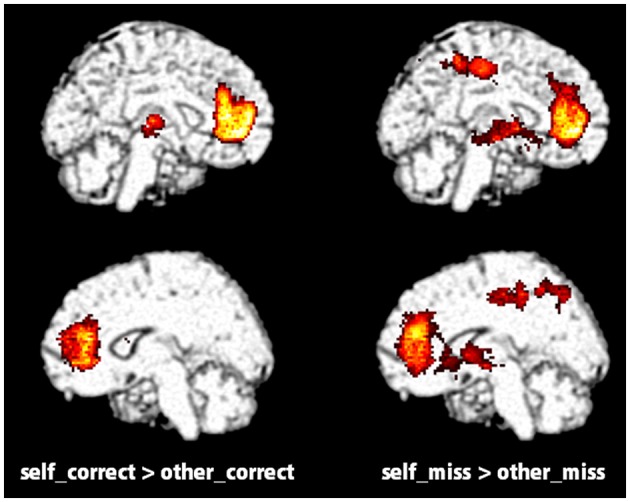
**Significantly activated regions distinguished by measuring the contrast between self (self_correct and self_miss) and other (other_correct and other_miss) conditions in the evaluation task (*p* < 0.05, FWE correction at the cluster level)**.

Table 3 and Figure 2 show brain activations according to the contrast between self/other and the correct/miss condition in the evaluation task using a random effect analysis (*p* < 0.05, FWE correction at the cluster level). Contrasts demonstrate differences in activation between the self and other; the main effects of self (self > other), self_correct, and self_miss conditions showed greater activation in relatively wide range of medial part of the prefrontal cortex (MPFC) containing the ventromedial prefrontal cortex (VMPFC) and anterior cingulate cortex (ACC) compared with their other counterparts. On the other hand, there was no significant difference between performance (correct vs. miss), the self_correct and self_miss, and the other_correct and other_miss conditions. Furthermore, we also could not find significant activation from both interactions, [(self_correct > self_miss) > (other_correct > other_miss)] and [(other_correct > other_miss) > (self_correct > self_miss)]. A summary of these findings by brain regions, Montreal Neurological Institute (MNI) coordinates, nearest Brodmann’s area (BA), and *Z* scores of each activated cluster are presented in Table 3.

**Table 3:** **MNI coordinates, approximate Brodmann’s area (BA), and *Z* scores of activated clusters indicated by the contrast between referential conditions (self_correct, self_miss, other_correct, and other_miss) and main effect (self vs. other)**.

Brain region	Cluster size	BA	*x*	*y*	*z*	*Z score*
***Self-correct > other-correct***
**anterior cingulate cortex**	**2763**	**24**	**−8**	**36**	**10**	**4.81**
		32	−6	50	0	4.80
Ventromedial prefrontal cortex		10	−10	54	8	4.61
**R insula**	**217**	**13**	**34**	**8**	**20**	**4.20**
R inferior frontal gyrus		44	50	−2	22	4.18
		9	44	4	24	3.31
**L thalamus**	**248**	**−**	**−12**	**−16**	**8**	**4.11**
			−18	−20	4	3.78
***Self-miss > other-miss***	
**anterior cingulate cortex**	**3200**	**10**	**−8**	**50**	**2**	**5.56**
		32	−6	34	0	4.95
Dorsomedial prefrontal cortex		9	12	42	20	4.86
**R thalamus**	**953**	**−**	**8**	**−2**	**4**	**4.39**
R subthalamic nucleus			6	−10	−6	4.35
L inferior frontal gyrus		47	−26	12	−14	4.22
**L paracentral lobe**	**1255**	**31**	**0**	**−24**	**50**	**4.38**
L middle frontal gyrus		6	−24	−16	44	4.16
L paracentral lobe		5	−16	−38	54	3.90
**R precuneus**	**432**	**7**	**16**	**−68**	**52**	**3.84**
R superior parietal lobe			22	−66	44	3.55
R temporal lobe		39	32	−62	26	3.53
**R middle temporal gyrus**	**164**	**37**	**54**	**−60**	**0**	**3.51**
R temporal lobe			44	−52	−6	3.29
***Self > other***	
**Medial prefrontal cortex**	**7386**	**10**	**−8**	**48**	**0**	**5.97**
Anterior cingulate cortex		24	−6	38	2	5.42
Medial prefrontal cortex		9	10	44	20	5.08
**Midcingulate cortex**	**13032**	**31**	**−8**	**−26**	**48**	**4.82**
L thalamus		−	−4	−14	−2	4.77
L inferior parietal lobe		40	−20	−40	54	4.63
**R cerebellum**	**1012**	**−**	**12**	**−64**	**−24**	**4.35**
			8	−54	−6	3.90
R fusiform gyrus			20	−56	−14	3.79
**L middle temporal gyrus**	**1168**	**37**	**−52**	**−58**	**0**	**4.26**
L cerebellum		−	−22	−62	−32	4.13
L middle temporal gyrus		37	−50	−68	4	3.93
**R inferior temporal gyrus**	**431**	**37**	**52**	**−52**	**−6**	**4.07**
		37	44	−50	−10	3.94
**R fusiform gyrus**	**266**	**36**	**38**	**−36**	**−12**	**4.05**
R hippocampus		−	34	−28	−6	4.92
			34	−20	−12	3.70

*Activation areas for all conditions were specified at p < 0.05 cluster level FWE corrected for multiple comparisons with the amplitude of voxels surviving at p < 0.001 uncorrected across the whole brain. Bold characters show voxels with maximum Z scores in the clusters*.

### fMRI Data (Recognition Task)

**Figure 3:**
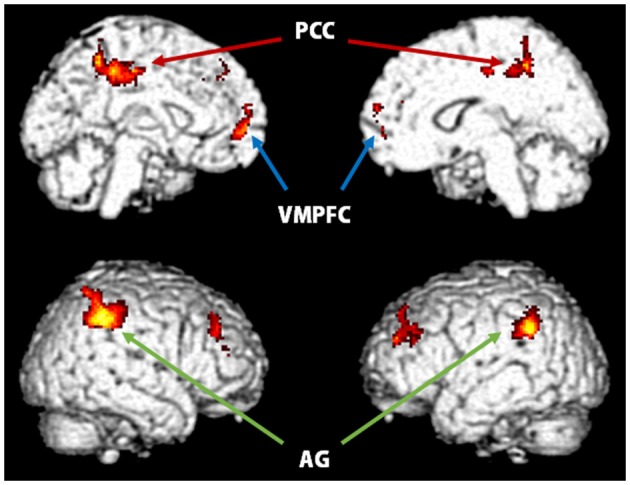
**Significantly activated regions distinguished by measuring the contrast between self_correct and other_correct conditions in the recognition task (*p* < 0.05, FWE correction at the cluster level)**. VMPFC, ventromedial prefrontal cortex; PCC, posterior cingulate cortex; AG, angular gyrus.

Table 4 and Figure 3 show brain activations according to the contrast between selfother and correct/miss recognition tasks using a random effect analysis (*p* < 0.05, FWE correction at the cluster level). Contrasts indicated that correct recognition for self-referenced words caused greater activation in ventral-to-middle part of MPFC, PCC and precuneus, bilateral angular gyrus (AG), and some other regions as compared with other-referenced words (Figure 3). On the other hand, there was no significant difference in other contrasts, other_correct > self_correct, self_miss > other_miss and other_miss > self_miss. And in common with the evaluation task, we could not find significant activation from interaction analysis.

**Table 4:** **MNI coordinates, approximate Brodmann’s area (BA), and *Z* scores of activated clusters indicated by the contrast between recognition conditions (self_correct, self_miss, other_correct, other_miss)**.

Brain region	Cluster size	BA	*x*	*y*	*z*	*Z_score_*
***self-correct > other-correct***	
**posterior cingulate cortex**	**629**	**31**	**−14**	**−34**	**44**	**4.94**
precuneus		7	−6	−46	50	3.89
posterior cingulate cortex		31	−8	−40	44	3.65
**R angular gyrus**	**774**	**40**	**66**	**−34**	**40**	**4.38**
R postcentral gyrus		2	62	−30	50	3.91
R inferior parietal lobe		40	60	−46	46	3.76
**L angular gyrus**	**329**	**40**	**−62**	**−42**	**32**	**4.21**
L supramarginal gyrus			−52	−48	36	4.11
L inferior parietal lobe			−58	−52	40	3.51
**ventromedial prefrontal cortex**	**175**	**10**	**−6**	**58**	**4**	**4.05**
			2	58	18	3.49
			−2	62	12	3.17
**L middle frontal gyrus**	**272**	**9**	**−26**	**32**	**32**	**4.01**
L superior frontal gyrus		8	−22	42	48	3.67
		10	−26	46	28	3.66
**R superior frontal gyrus**	**164**	**9**	**34**	**36**	**34**	**3.94**
R middle frontal gyrus		10	28	42	22	3.70
		8	36	34	44	3.39

*Activation areas for all conditions were specified at p < 0.05 cluster level FWE corrected for multiple comparisons with the amplitude of voxels surviving at p < 0.001 uncorrected across the whole brain. Bold characters show voxels with maximum Z scores in the clusters*.

## Discussion

In this experiment, RTs for the self- and other-referential tasks did not show a significant main effect, but revealed a significant interaction between condition (self vs. other) and performance (correct vs. miss). The *post hoc* analysis indicated that mean RTs during the self_correct were marginally faster than those during self_miss and other_correct. This result would indicate that a relatively longer RT for correct recognition was required when words were referred to other than self. Conversely, correct recognition for the self-referenced words would involve in other factors made RT shorter, for example, self-descriptiveness (Kuiper and Rogers, 1979). However, because we could not find significant differences between any conditions from *post hoc* analysis, it is difficult to make a strong claim about differences of RTs. In any case, participants were more accurate at recognizing whether trait adjectives referred to themselves than if a trait had been applied to another (Table 2), suggesting that SRE occurred.

The fMRI data from the evaluation task (Figure 2; Table 3) showed that the contrast between self_correct and other_correct and between self_miss and other_miss was significant in VMPFC (BA10), ACC (BA24, 32) and some other regions regardless of whether or not the words were retrieved correctly in later recognition tasks. These results are consistent with previous studies that indicated a greater activation in VMPFC in self-reference tasks. Those studies implied that VMPFC was involved in the representation of self-referential stimuli and constituted a part of self-related brain area called the cortical midline structure (CMS; Northoff and Bermpohl, 2004; Heatherton et al., 2006). This region also has been known to be one of important part of the default mode network (DMN) that is indicated to relate internal self-related processing (Gusnard et al., 2001; Buckner et al., 2008; Sheline et al., 2009; Spreng and Grady, 2010; Salomon et al., 2014). Kelley et al. (2002) and Yoshimura et al. (2009) used the above mentioned trait-adjective judgment task in an fMRI study to demonstrate that VMPFC was selectively activated in the self-referential condition. Furthermore, Denny et al. (2012) performed a meta-analysis of neuroimaging studies and reported that self-related judgments were associated with relatively ventral MPFC (BA 10) and anterior paracingulate cortex (BA 32), whereas other-related judgments were associated with relatively dorsal MPFC (BA 8 and 9). Our data would support this theory. From the standpoint of a cognitive and neural basis for SRE, however, we observed that activation of VMPFC and other regions in the self-reference task occurred regardless of recognition. Furthermore, the results of the contrast between self_correct and self_miss did not show any significant differences. It is difficult to draw any obvious conclusions for a relationship between VMPFC activation and SRE only from these results, but there is a possibility that it cannot provide an adequate explanation for the neural basis of SRE if only the evaluation phase is used.

In the recognition task, the results of contrast between the four conditions showed significant differences, particularly between self_correct and other_correct (Figure 3; Table 4). This demonstrates that ventral-to-middle MPFC (BA10), PCC, bilateral AG, and some other regions have a greater activation for the correct recognition of words that refer to the self. These regions did not show significantly different activations when the recognition task found a miss, therefore, these regions might be involved in processes specific to the correct recognition of words referring to the self by the self-reference task. However, there was no significant activation from interaction analysis, such as [(self_correct > self_miss) > (other_correct > other_miss)]. We consider that this result is due to the effect of biased number of fMRI events, especially a lack of self_miss condition. In this experiment, participants’ performances of self-referential task were generally high. For example, a participant got almost all the answers correct in the self-referential task, then there was only one event for self_miss condition. Like this, because of the difference in the number of events across conditions, it would be difficult to obtain appropriate results.

Greater VMPFC activation in the self_correct condition only would indicate that self-representation is re-activated under the retrieval of self-referenced words in the recognition task. Our results show that some information networks linked with the self-representation were activated once the words were referred to the self in the encoding phase, and worked as efficient internal cues in the re-activation of the words in the retrieval phase. On the other hand, it was relatively difficult for words that referred to a distant other once activated with the other-representation to provide efficient cues in re-activation. Some studies have proposed that one of the reasons for SRE is that self-representation provides richer and more organized internal cues and facilitates the retrieval of self-referenced items during the retrieval processes (Bellezza, 1984; Klein and Loftus, 1988). The greater VMPFC activation in the recognition phase supports this idea.

MPFC is also a region important for successful episodic memory retrieval (Wagner et al., 2005; Buckner et al., 2008). Huijbers et al. (2010) used a continuous recognition task with varying retrieval delays to show that MPFC activation increased with longer memory delays when recognition decisions become more dependent on hippocampus activity. Leshikar and Duarte (2012) and Morel et al. (2014) also found that activity in MPFC contributes to a self-related memory benefit. Our results confirm that MPFC is involved in retrieving memory processes, especially for self-referenced items rather than a distant other.

The PCC is also included in the CMS and may be involved in integrating information related to the self (Northoff and Bermpohl, 2004). This region, comprised in the DMN, has been reported to elicit significant activation in previous studies that used self-referential paradigms (Zysset et al., 2002; Schmitz et al., 2004; Seger et al., 2004; Yaoi et al., 2009) and known to be involved in the retrieval of episodic or autobiographical memory (Maguire and Mummery, 1999; Hassabis et al., 2007). Furthermore, the bilateral AG also showed greater activation for the self_correct over the other_correct condition. It has been suggested that the AG is also involved in encoding and retrieving processes for episodic memory (Maguire and Mummery, 1999; Wagner et al., 2005). Although participants could correctly recognize words in both the self_correct and other_correct conditions, we show here differential activations in these memory-related brain regions. We suggest that stronger memory traces of words referring to the self remained in the recognition phase and that this property has some relationship with the better recognition performance in the recognition task. Using a self-referential paradigm, Macrae et al. (2004) revealed that activity in MPFC can predict both subsequent memory performance and judgments of self-relevance. Furthermore, Morel et al. (2014) found that the self condition shows greater activation in VMPFC extending to ACC, hippocampus, and some other regions but only in the encoding phase, not in the retrieval phase, relative to control semantic conditions. Our results provide further evidence that the greater activation of MPFC, PCC and bilateral AG is involved in retrieving memory processes of self-referenced items compared with others.

To uncover further details about the cognitive and neural basis of SRE, several unresolved issues remain. We compared the self only with a distant other referential condition. Thus, we could not discriminate whether increased activations for the self in the evaluation or recognition tasks would remain significantly higher for other targets, like a close other (for example, a parent or friend). Furthermore, in the recognition task, participants did not judge their confidence in the memory of each word. Therefore, we could not conclude whether memory traces of the words referring to the self were actually stronger than others. As a result, the recognition performance of the self-referential condition became better. To clarify whether activation of the parahippocampal cortex or AG reflects the strength of memory in the recognition process, analyses based on the memory confidence test assessed by a Remember-Know paradigm should be considered (Tulving, 1985; Gardiner, 1988). For example, Kim and Cabeza (2009) investigated whether, and to what extent, brain regions involved in high- vs. low-confidence recognition for words overlap or remain separate from each other. They found greater activation in the hippocampus and ACC and PCC associated with words recognized with high-confidence, and activation in the dorsal precuneus and dorsolateral and posterior prefrontal cortex associated with words recognized with low-confidence. This approach could clarify the relationship between the activation of memory-related brain regions and recognition/recall processes in more detail by including memory confidence of words that refer to the self and others in the recognition phase as part of the assessment. Additionally, one factor that could influence our results is culture. Previous studies indicated that there were cultural differences, not only behavioral but also neural, in self-referential processing (Zhu et al., 2007; Korn et al., 2014; Ma et al., 2014). SRE is known to be relatively robust across cultures, but it is possible to show different results between participants from different cultural backgrounds (for example, Westerner vs. East Asian) when they perform referential and recognition tasks, especially in the comparison between self and close-other, for example, a parent or friend.

## Conclusion

Using event-related fMRI, we measured brain activity during processing of references to the self and a distant other, and of recognitions for self and other referenced words. Behavioral data confirm that SRE occurred during this experiment. The fMRI results from the evaluation task indicated that VMPFC activation was more closely associated with self-referential processing, consistent with previous studies. Results from the recognition task showed that correct recognition of the self-referenced words coincided with greater activation in VMPFC, PCC and bilateral AG. These data provide evidence that the self-referenced words evoked greater activation in the self-related region (VMPFC) and memory-related regions (PCC and AG) relative to another person in the retrieval phase, and that the words remained as a stronger memory trace that supports recognition.

## Conflict of Interest Statement

The authors declare that the research was conducted in the absence of any commercial or financial relationships that could be construed as a potential conflict of interest.
